# Piperlongumine Inhibits Thioredoxin Reductase 1 by Targeting Selenocysteine Residues and Sensitizes Cancer Cells to Erastin

**DOI:** 10.3390/antiox11040710

**Published:** 2022-04-04

**Authors:** Yijia Yang, Shibo Sun, Weiping Xu, Yue Zhang, Rui Yang, Kun Ma, Jie Zhang, Jianqiang Xu

**Affiliations:** 1 School of Life and Pharmaceutical Sciences (LPS), Panjin Institute of Industrial Technology (PIIT), Liaoning Key Laboratory of Chemical Additive Synthesis and Separation (CASS), Dalian University of Technology, Panjin 124221, China; clnyyj@mail.dlut.edu.cn (Y.Y.); sunshibo@dlut.edu.cn (S.S.); m15663671275_1@mail.dlut.edu.cn (Y.Z.); yangruirui@mail.dlut.edu.cn (R.Y.); makunonline@dlut.edu.cn (K.M.); 2 School of Ocean Science and Technology (OST), Key Laboratory of Industrial Ecology and Environmental Engineering (MOE), Dalian University of Technology, Panjin 124221, China; weiping.xu@dlut.edu.cn; 3 Department of Cell and Molecular Pharmacology and Experimental Therapeutics, Medical University of South Carolina, Charleston, SC 29425, USA; zhajie@musc.edu

**Keywords:** piperlongumine, thioredoxin reductase 1, erastin, glutathione, selenocysteine, CB-839

## Abstract

Piperlongumine, a natural alkaloid substance extracted from the fruit of the long pepper (*Piper longum Linn*.), is known to inhibit the cytosolic thioredoxin reductase (TXNRD1 or TrxR1) and selectively kill cancer cells. However, the details and mechanism of the inhibition by piperlongumine against TXNRD1 remain unclear. In this study, based on the classical DTNB reducing assay, irreversible inhibition of recombinant TXNRD1 by piperlongumine was found and showed an apparent *k*_inact_ value of 0.206 × 10^−3^ µM^−1^ min^−1^. Meanwhile, compared with the wild-type TXNRD1 (-GCUG), the UGA-truncated form (-GC) of TXNRD1 was resistant to piperlongumine, suggesting the preferential target of piperlongumine is the selenol (-SeH) at the C-terminal redox motif of the enzyme. Interestingly, the high concentration of piperlongumine-inhibited TXNRD1 showed that its Sec-dependent activity is decayed but its intrinsic NADPH oxidase activity is retained. Furthermore, piperlongumine did not induce ferroptosis in HCT116 cells at 10 µM, whereas significantly promoted erastin-induced lipid oxidation, which could be alleviated by supplying glutathione (GSH) or N-acetyl L-cysteine (NAC). However, restricting GSH synthesis by inhibiting glutaminase (GLS) using the small molecule inhibitor CB-839 only slightly enhanced erastin-induced cell death. Taken together, this study elucidates the molecular mechanism of the antitumor capacity of piperlongumine by targeting TXNRD1 and reveals the potential possibility of inhibiting TXNRD1 to strengthen cancer cells’ ferroptosis.

## 1. Introduction

Mammalian thioredoxin reductase 1 (TXNRD1 or TrxR1) is generally regarded as a homodimeric selenoenzyme involved in antioxidant defenses [1]. Besides, TXNRD1 is actually associated with normal cell survival, division, migration, and proliferation; however, it has been reported to be up-regulated in numerous cancer cells. Since cancer cells possess high levels of endogenous reactive oxygen species (ROS) and are more sensitive to redox imbalance than normal cells, targeting TXNRD1 to disturb cell redox balance has been exploited as a promising strategy for cancer therapy [2,3]. 

Each subunit of TXNRD1 contains a surface-exposed hyperreactive selenocysteine (Sec, U) residue, which is essential for the functional reduction of its physiological substrate thioredoxin 1 (TXN1 or Trx1) or thioredoxin-related protein of 14 kDa (TRP14) [4,5]. In order to target the Sec residue of TXNRD1 to induce cancer cell death, various electrophilic small molecules have been screened, designed, and developed so far [6,7,8]. The detailed interactions of small molecule inhibitors or other plant-resourced pharmaceutical components to target the active sites of TXNRD1 are in need of in-depth investigations.

Piperlongumine has been reported as a naturally occurring alkaloid substance with potent anti-tumor activities in vitro and in vivo [9]. Piperlongumine selectively kills tumor cells by generating cellular ROS, and a slew of key enzymes have been identified as targets of piperlongumine, such as NF-κB, STAT3, and TXNRD1 as previously studied [10,11,12,13]. However, the details of piperlongumine as a promising pro-oxidative drug that inhibits TXNRD1 and subsequently stimulates ROS generation and conveys redox modulation remain elusive. Meanwhile, whether the antitumor activity of piperlongumine is associated with TXNRD1 is so far unclear.

Ferroptosis, an iron-dependent necroptosis caused by phospholipids oxidation, is a targetable vulnerability in cancer. The SLC7A11–GSH–GPX4 axis acts as one of the critical ferroptosis regulators [14]. Erastin inhibits the cystine-glutamate antiporter SCL7A11, which depletes cellular GSH, leading to lipid peroxidation and ferroptosis [15]. TXN1/TXNRD1 facilitates the synthesis of GSH, being involved in the reduction of cystine to cysteine, which is thought to be a negative regulator of ferroptosis [5,16]. Meanwhile, the TXN system quenches cellular H_2_O_2_ via peroxidases, which is essential for the Fenton reaction to produce hydroxyl radicals, suggesting the potential role of TXNRD1 in ferroptosis suppression.

To gain insights into the influence of piperlongumine on TXNRD1, an attempt was made to investigate its inhibition using recombinant selenoenzyme TXNRD1 and cultured cancer cell lines to explain how the interaction contributes to the antitumor activity of piperlongumine. In this study, piperlongumine was found to irreversibly inhibit TXNRD1 activity, the underlying mechanism of which may be by targeting the Sec^498^ residue and thus acting as a hyper-reactive pro-oxidant more than SecTRAPs (selenium compromised thioredoxin reductase-derived apoptotic proteins) [17,18]. Notably, the inhibition of TXNRD1 by piperlongumine as well as TXNRD1 inhibitors auranofin and TRi-1, sensitize cancer cells to erastin-induced cell death. Meanwhile, pharmacologic inhibition of glutaminase (GLS) by CB-839 also slightly boosts the cytotoxicity of erastin. This study clarifies the mechanism of the antitumor activity of piperlongumine by targeting TXNRD1 and also contributes to the further application of piperlongumine in combination with ferroptosis inducers in clinical chemotherapy.

## 2. Materials and Methods

### 2.1. Materials and Reagents

*Escherichia coli* BL21 (DE3) *gor*¯ host strain was generously provided by Professor Arne Holmgren, Karolinska Institutet (Sweden). Plasmids pET-TRS_TER_ and pSUABC were kindly provided by Professor Elias S.J. Arnér at Karolinska Institutet (Stockholm, Sweden) & National Institute of Oncology (Budapest, Hungary). Recombinant rat TXNRD1 and its mutants were prepared according to the previous method [19,20] and generally stored in 50 mM TE buffer (containing 2 mM EDTA, pH 7.5). 5-hydroxy-1,4-naphthoquinone (juglone), 9,10-phenanthrene quinone (9,10 PQ), and 5,5′-dithiobis-(2-nitrobenzoic acid) (DTNB) were purchased from Sigma-Aldrich (St. Louis, MO, USA). Nicotinamide adenine dinucleotide phosphate (NADPH), piperlongumine, erastin, deferoxamine mesylate (DFO), deferasirox (DFX), ferrostatin-1 (Fer-1), liproxstatin-1 (Lip-1) and CB-839 were obtained from Yuanye Biotechnology (Shanghai, China). Dulbecco’s Modified Eagle’s Medium (DMEM) was supplied from Gibco (Carlsbad, CA, USA), heat-inactivated fetal bovine serum (FBS) was sourced from PAN (Aidenbach, Germany), and McCoy 5A medium was purchased from Procell (Wuhan, China). Cell Counting Kit-8 (CCK-8) and BCA protein concentration kit were purchased from Beyotime Biotechnology (Shanghai, China). ÄKTA Start^TM^ protein purification workstation, 2′,5′-ADP Sepharose^TM^ affinity column, prepacked 16/60 Sephacryl^TM^ S-300 HR column, and NAP^TM^-5 desalting columns were all purchased from Cytiva (Uppsala, Sweden). All enzyme-based assays were performed in a 50 mM TE buffer containing 2 mM EDTA (pH 7.5) at room temperature unless stated else.

### 2.2. Cell Culture

Human lung cancer cells (A549), human breast cancer cells (MCF-7), and human liver cancer cells (HepG2) were grown in DMEM (supplemented with 10% FBS, 100 U/mL penicillin, and 100 mg/mL streptomycin). Human colon cancer cells (HCT116) were grown in McCoy 5A medium supplemented with 10% FBS, 100 U/mL penicillin, and 100 mg/mL streptomycin, in a humidified incubator (Heal Force, Shanghai, China) with an atmosphere of 5% CO_2_ and a temperature of 37 °C.

### 2.3. Cell Viability Assay

The cell viability was detected with the Cell Counting Kit-8 (CCK-8) according to the manufacturer’s instructions. Briefly, cells were seeded into 96-well plates at the density of 5000 cells per well in a final volume of 100 µL and incubated in the atmosphere overnight [21,22]. The next day, cells were treated with different dosages compounds as indicated. After incubation, 10 µL of CCK-8 reagent was added, and incubation was continued at 37 °C for 2 h. Finally, the absorbance was measured at 450 nm and the reference wavelength was measured at 600 nm. The untreated group was used as the control.

### 2.4. Thioredoxin Reductase Activity Assays

Thioredoxin reductase activities were determined using the human TXN1-coupled insulin reducing assay, DTNB reducing assay, and 9,10 PQ reducing assay, respectively. In the DTNB reducing assay, the final reaction mixture contained 2.5 mM DTNB and 300 µM NADPH, and 10 nM wild-type TXNRD1 or more TXNRD1 mutant variants (30 nM for GCCG and 100 nM for other mutants) as indicated, in 50 mM TE buffer (pH 7.5), and the enzyme activity was calculated by the TNB¯ formation at 412 nm (ε_TNB¯_ = 13,600 M^−1^ cm^−1^). In the TXN1-coupled insulin reducing assay, the final mixture contained 50 nM TXNRD1, 10 µM human TXN1, 160 µM insulin, and 300 µM NADPH in 50 mM TE buffer (pH 7.5). In the 9,10 PQ reducing assay, the final mixture contained 30 nM wild-type TXNRD1, 30 µM 9,10 PQ, and 200 µM NADPH in 50 mM TE buffer (pH 7.5). The enzymatic activity of human TXN1-coupled insulin reduction and 9,10 PQ reduction was calculated by the NADPH oxidation at 340 nm (ε_NADPH_ = 6200 M^−1^ cm^−1^). All reactions were performed in an Infinite 200 PRO plate reader (Tecan, Männedorf, Switzerland) at 25 °C. The same reaction mixture lacking the enzyme was used as a reference.

### 2.5. NADPH Oxidase Activity Assay

The oxidation of NADPH was determined by juglone reduction according to the previous reports [23,24]. In brief, the master mixture contained 30 µM juglone and 200 µM NADPH in 50 mM TE buffer (pH 7.5). The NADPH oxidase activity was calculated by the NADPH oxidation at 340 nm (ε_NADPH_ = 6200 M^−1^ cm^−1^) using an Infinite 200 PRO Plate Reader (Tecan, USA) at 25 °C. The same reaction mixture lacking the enzyme was used as a reference.

### 2.6. Cellular TXNRD Activity Assay

Cellular TXNRD activity was determined using a TXN1-coupled end-point insulin assay, as previously described [25,26,27]. Cells were seeded into 6-well plates at 400,000 cells per well. Twelve hours later, compounds were added at indicated concentrations and incubated for a period of time (2.5 h and 5 h) at 37 °C. Then, the cells were lysed on ice with RIPA lysis buffer (Beyotime, Shanghai, China) for 10 min and centrifuged at 18,000 rpm for 20 min. Protein concentrations of samples were determined using the BCA kit (Beyotime, Shanghai, China) and BSA was used as standard protein. In brief, an appropriate amount of cell lysate was added into a master mixture containing 80 mM Hepes buffer, pH 7.5, 15 μM TXN1, 300 μM insulin, 660 μM NADPH, and 3 mM EDTA. A reaction mixture without TXN1 was used as a background control. Samples were incubated at 37 °C for 30 min. Subsequently, 6.0 M guanidine hydrochloride containing 1 mM DTNB, 20 mM EDTA was added to each well, and an endpoint at OD412 was measured. TXNRD activities of cell lysates were normalized to protein concentration for accurate comparison.

### 2.7. Glutathione Reductase Activity Assay

Glutathione reductase (GR) activity was determined using the oxidized glutathione (GSSG) as a substrate [28]. The master mixture (200 µL) contained 1 mM GSSG, 2 nM yeast GR, and 200 µM NADPH. GR activity was calculated by following the NADPH oxidation at an absorbance of 340 nm (ε_NADPH_ = 6200 M^−1^ cm^−1^) using an Infinite 200 PRO plate reader (Tecan, Männedorf, Switzerland) at 25 °C. The same reaction mixture lacking the enzyme was used as a reference.

### 2.8. Cellualr GSH Content Determination

Cellular GSH levels were determined by assaying the total thiols. In brief, cell lysis solution was reacted with a master mixture containing 6.0 M guanidine hydrochloride, 1 mM DTNB and 20 mM EDTA for 5 min, and an endpoint at OD412 was measured. Total thiols of cell lysates were normalized to protein concentration for accurate comparison.

### 2.9. Cellular ROS Levels Determination

Cellular ROS levels were assayed by DCFH-DA. Cells were seeded into a black 96-well plate at the density of 5000 cells per well. After indicated incubation, cells were incubated with 10 µM DCFH-DA at 37 °C for 60 min and washed with PBS three times. Cellular ROS levels were then assayed using an Infinite 200 PRO plate reader (Tecan, Männedorf, Switzerland) with excitation at 488 nm and emission at 525 nm.

### 2.10. Lipid Oxidation Assays

Lipid oxidation was assayed by flow cytometry using BODIPY^TM^ 581/591 C11. In brief, cells were incubated with 10 µM BODIPY^TM^ 581/591 C11 at 37 °C for 60 min and washed with PBS three times. Cells were then digested to a single-cell suspension in 5% FBS medium for the FACS Calibur flow cytometry detection (BD, Franklin Lakes, NJ, USA).

### 2.11. Statistics Analysis

All experiments were performed in triplicate and the data were presented as the mean ± S.E.M. Statistical differences between the two groups were analyzed by the Student’s *t*-test. Comparisons among multiple groups were statistically assessed by one-way analysis of variance (ANOVA) and followed by a post hoc Scheffe test. The significant differences between groups were defined as * *p* < 0.05, ** *p* < 0.01, *** *p* < 0.001, and n.s. means not significant.

## 3. Results

### 3.1. Piperlongumine Inhibits TXNRD1 in a Dose-Dependent Manner

**Figure 1:**
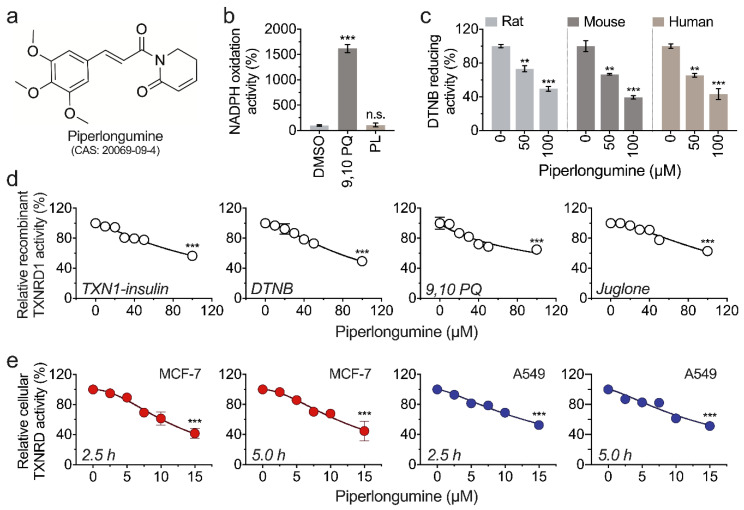
Piperlongumine inhibits TXNRD1 activity. (**a**) The chemical structure of piperlongumine (PL). (**b**) Piperlongumine is not a proper substrate of TXNRD1. The NADPH oxidation activity of 30 nM TXNRD1 in the presence of 200 μM NADPH and 30 μM piperlongumine or 9,10 PQ (DMSO as control) were measured, respectively. (**c**) Piperlongumine inhibits three species of recombinant TXNRD1. Human TXNRD1 (0.4 μM), rat TXNRD1 (0.2 μM), and mouse TXNRD1 (0.3 μM) were incubated with piperlongumine for 30 min and the enzyme activities were measured by DTNB reducing activity assay. (**d**) Inhibition of rat TXNRD1 with different substrates by piperlongumine. Rat TXNRD1 was incubated with piperlongumine for 30 min, and the retained enzyme activity was measured by TXN1-coupled insulin-reducing activity assay, DTNB-reducing activity assay, 9,10 PQ-reducing activity assay, and juglone-reducing activity assay, respectively. (**e**) Inhibition of cellular TXNRD activity by piperlongumine. MCF-7 and A549 cells were treated with piperlongumine for 2.5 h and 5 h. The cellular TXNRD activity was measured by TXN1-coupled end-point insulin assay, and the activity was normalized to protein content.

We first investigated whether piperlongumine could accept electrons from TXNRD1. As shown in Figure 1a, there was no difference between piperlongumine and DMSO in TXNRD1-mediated NADPH oxidation activity. In contrast, 9,10 PQ, a substrate of TXNRD1, showed an approximately 10-fold greater activity compared with piperlongumine, suggesting piperlongumine is not a proper substrate of TXNRD1 (Figure 1b). In the previous study, recombinant rat TXNRD1 was usually used as a model in place of human TXNRD1 in recombinant TXNRD1-related experiments. In this study, piperlongumine presented a similar inhibitory effect on human, rat, and mouse TXNRD1s (Figure 1c). Besides, we found that piperlongumine inhibits TXNRD1 activity on either TXN1-coupled insulin reduction, DTNB reduction, or 9,10 PQ reduction, and the inhibition is in a dose-dependent manner. After being incubated with 100 µM piperlongumine for 30 min, the remaining activity of TXNRD1 was approximately 50% in reducing these substrates (Figure 1d). Meanwhile, the cellular TXNRD activity was also inhibited by piperlongumine in a dose-dependent manner in MCF-7 and A549 cells (Figure 1e). Notably, a strong inhibition of TXNRD activity was observed in cancer cell lines by piperlongumine compared with the purified enzyme; however, the reason for this phenotype is currently unexplained.

### 3.2. Piperlongumine Irreversibly Inhibits TXNRD1 In Vitro

**Figure 2:**
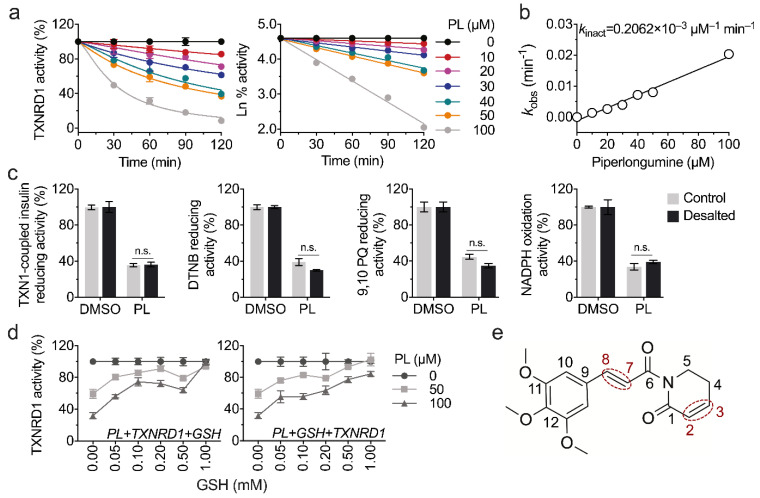
Time-dependent irreversible inhibition of piperlongumine on TXNRD1. (**a**) Piperlongumine inhibits TXNRD1 in a time-dependent manner. TXNRD1 was incubated with different concentrations of piperlongumine for the indicated time. Enzyme activity was measured by DTNB-reducing activity assay. The remaining activity was transformed into the natural logarithm and linear fitted in the right panel. (**b**) Second inhibition rate of piperlongumine on TXNRD1. The slope derived from panel a (right) was plotted versus the concentrations of piperlongumine, and the *k*_incat_ was calculated from the slope. (**c**) Irreversible inhibition of piperlongumine on TXNRD1. TXNRD1 was incubated with 100 μM piperlongumine for 60 min. Afterwards, the samples were subjected to NAP^TM^-5 desalting columns and eluted with fresh TE buffer. Finally, the remaining activities of TXNRD1 in the eluted fractions were measured by using various substrates accordingly. (**d**) GSH protects/rescues the activity loss of recombinant TXNRD1. In the left panel, the mixture contained GSH, piperlongumine, TXNRD1, and NADPH. In the right panel, GSH was pre-incubated with piperlongumine for 10 min and then TXNRD1 and NADPH were added to the mixture. After being incubated for 60 min, the TXNRD1 activity was determined by 9,10 PQ-reducing assay. (**e**) Two reactive α,β-unsaturated olefins of piperlongumine.

We found that the inhibition of piperlongumine on TXNRD1 is time dependent, as shown in Figure 2a. The *k*_inact_ value is 0.206 × 10^–3^ μM^−1^ min^−1^ in the classic DTNB-reducing activity assay (Figure 2b). To validate whether the inhibition is irreversible or not, we incubated the TXNRD1 with piperlongumine for 60 min and then subjected it to a NAP^TM^-5 desalting column to remove free compounds. The remaining enzyme activity was assayed in the TXN1-coupled insulin-reducing assay, DTNB-reducing assay, and 9,10 PQ-reducing assay. Regarding the remaining enzyme activities, there was no significant difference between the desalted and undesalted samples, suggesting an irreversible inhibition of piperlongumine on TXNRD1 (Figure 2c). What is more, considering the results that the inhibition of TXNRD1 by electrophiles is usually through the Michael addition, we tried to verify the inhibition of TXNRD1 by piperlongumine. As shown in Figure 2d,e, a high amount of GSH could fully protect the TXNRD1 activity from piperlongumine.

### 3.3. Piperlongumine Targets the Sec^498^ of TXNRD1 and Converts the Enzyme to NADPH Oxidase

**Figure 3:**
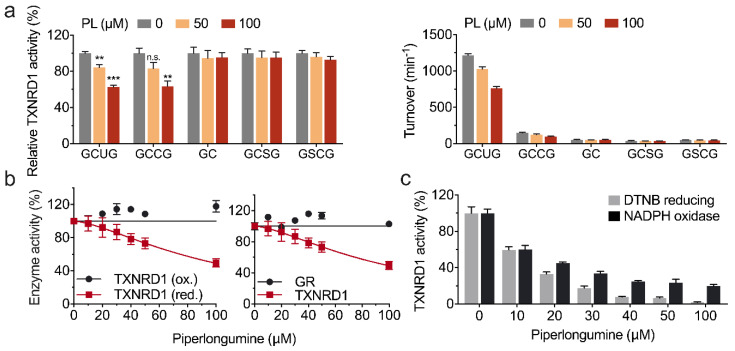
Piperlongumine targets the Sec^498^ or Cys^498^ residue of TXNRD1 and converts the enzyme to NADPH oxidase. (**a**) Piperlongumine inhibits TXNRD1 and its Sec-to-Cys mutant rather than other Sec-deficiency mutants. NADPH pre-reduced wild-type TXNRD1 (10 nM) and TXNRD1 variant mutants (30 nM for GCCG and 100 nM for other mutants) were incubated with piperlongumine for 30 min, respectively. TXNRD1 activities were determined by using DTNB-reducing assay, and the relative activity was shown in left panel and the absolute turnover was shown in right panel. (**b**) Piperlongumine has no inhibitory effect on oxidized form TXNRD1 and GR activity. 100 μM NADPH pre-reduced TXNRD1 (red.) or non-pre-reduced TXNRD1 (ox.) or GR was incubated with piperlongumine at various concentrations for 30 min. GR activity was determined using GSSG as substrate in the presence of 200 µM NADPH. The TXNRD1 activities were determined by using DTNB-reducing assay. (**c**) High concentration of piperlongumine inhibits TXNRD1 Sec-dependent activity but remains NADPH oxidation activity. NADPH oxidation activity was measured by following the NADPH oxidation at OD340 nm using juglone as substrate.

To identify which residues were targeted by piperlongumine on TXNRD1, TXNRD1 and its mutant variants were incubated with piperlongumine for 30 min separately and assayed for the residual activity. The result showed that both wild-type TXNRD1 and its Sec-to-Cys mutant (GCCG or U498C) were inhibited by piperlongumine, while the other mutants were insensitive to piperlongumine (Figure 3a). As the Sec^498^ residue of TXNRD1 is surface exposed and more susceptible to electrophilic reagents, we speculated that piperlongumine modified the Sec^498^ (or Cys^498^) of TXNRD1 by the Michael addition reaction, which was also consistent with the results that the oxidized TXNRD1 and glutathione reductase (GR) were not inhibited by piperlongumine (Figure 3b). Furthermore, once the enzyme activity of TXNRD1 was fully inhibited by piperlongumine, indicating the formation of SecTRAPs, it was consistent with the fact that the resulting product lost its antioxidants activity but still retained its NADPH oxidase activity (Figure 3c).

### 3.4. Piperlongumine Induces ROS-Dependent Cancer Cell Death but Not Ferroptosis

**Figure 4:**
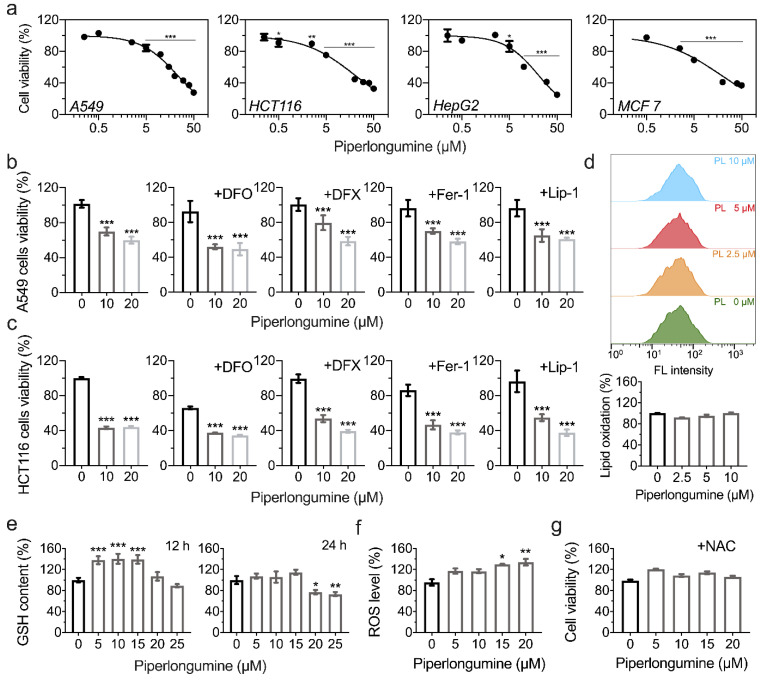
Piperlongumine induces cancer cells death. (**a**) Cytotoxicity of piperlongumine on cancer cell lines. A549, HCT116, HepG2, and MCF-7 cells were treated with piperlongumine in the range of 0 to 50 µM for 24 h, and then the cell viabilities were determined by the CCK-8 assay. (**b**,**c**) Ferroptosis inhibitors do not rescue the cell death caused by piperlongumine in A549 and HCT116 cells. (**d**) Piperlongumine does not induce ferroptosis in HCT116 cells. The lipid oxidation induced by piperlongumine was measured by BODIPY^TM^ 581/591 C11 using flow cytometry. (**e**) GSH contents in HCT116 cells upon piperlongumine treatment. (**f**) Increased ROS levels caused by piperlongumine in A549 cells. ROS level was determined by fluorescence probe DCFH-DA and monitored by a fluorescence microplate reader. (**g**) NAC protected A549 cells from piperlongumine.

Ferroptosis is a newly observed programmed necrotic cell death caused by lipid peroxidation [29]. We next tested whether the cell death caused by piperlongumine is ferroptosis. As shown in Figure 4a, piperlongumine showed cytotoxicity in A549, HCT116, MCF-7, and HepG2 cells. To confirm whether piperlongumine-induced cell death is ferroptosis, we introduced four ferroptosis inhibitors in the incubation system, including two iron chelators, DFO (50 µM) and DFX (25 µM), and two ferroptosis inhibitors, Fer-1 (2 µM) and Lip-1 (1 µM). However, these inhibitors did not rescue the cell viability loss from piperlongumine (Figure 4b,c). Furthermore, we did not observe lipid oxidation by BODIPY^TM^ 581/591 C11 stain (Figure 4d), indicating that piperlongumine does not induce cell ferroptosis, especially under 10 µM. What is more, we found that piperlongumine decreased the cellular GSH contents (Figure 4e). An increased ROS level was observed upon piperlongumine treatment in A549 cells (Figure 4f), and 1 mM of NAC could alleviate the cytotoxicity of piperlongumine (Figure 4g), indicating a ROS-dependent cell death caused by piperlongumine, whereas it is not ferroptosis under low concentrations of piperlongumine.

### 3.5. Piperlongumine Enhances Erastin-Induced Cancer Cells Death

**Figure 5:**
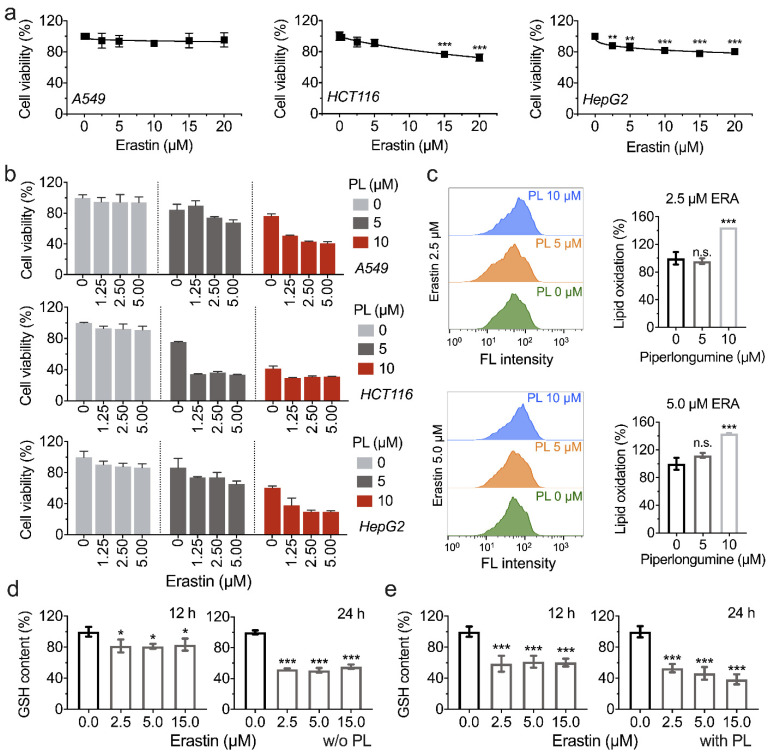
Piperlongumine may enhance erastin-induced cancer cell death. (**a**) Cytotoxicity of erastin on cancer cell lines. A549, HCT116, and HepG2 cells were treated with erastin in the range of 0 to 20 µM at various concentrations for 24 h and then the cell viabilities were measured by the CCK-8 assay. (**b**) Synergistic effect of erastin and piperlongumine. A549, HCT116, and HepG2 cells were incubated with both piperlongumine and erastin at the indicated concentrations for 24 h. Afterwards, the cell viability was measured by using the CCK-8 assay. (**c**) Piperlongumine forces erastin-induced ferroptosis. HCT116 cells were treated with piperlongumine and erastin as indicated, and the lipid oxidation was measured by BODIPY^TM^ 581/591 C11 using flow cytometry. (**d**,**e**) GSH content in HCT116 cells upon erastin treatment with or without 5 µM piperlongumine.

Ferroptosis inducers can be used as a promising therapy to promote cancer cell ferroptosis [14]. Erastin, known as a ferroptosis inducer, and identified as an inhibitor of cysteine/glutamate antiporter and glutathione synthesis, was used in this experiment in combination with piperlongumine to treat cancer cells. To validate the application potential of piperlongumine in cancer therapy by enhancing ferroptosis, a combination of piperlongumine with erastin was investigated for its anti-tumor activity. The lethality of erastin in A549, HCT116, and HepG2 cell lines was first evaluated, as shown in Figure 5a. Importantly, A549 cells showed resistance upon erastin treatment, indicating the endogenous antioxidants system protected cells from GSH depletion, which was caused by the pharmacological inhibition of system Xc¯ by erastin. Since the GSH system and TXN system are the prominent cellular redox regulation systems, an attempt was made to inhibit TXNRD1 activity by piperlongumine to enhance erastin-induced cell death in cancer cells. Surprisingly, as shown in Figure 5b, 5 μM (or 10 μM) piperlongumine increased cell death upon erastin treatment in A549, HCT116, and HepG2 cell lines, respectively, suggesting the synergistic effect of piperlongumine and erastin in treating cancer cells, and erastin-induced lipid oxidation was also increased by piperlongumine (Figure 5c). To clarify the mechanism of piperlongumine forced erastin-induced cell death, the cellular GSH content was assayed. We found that piperlongumine significantly enhanced erastin-induced GSH depletion (Figure 5d,e).

### 3.6. Inhibition of TXNRD1 Activity Sensitizes Cancer Cell to GSH Depletion

**Figure 6:**
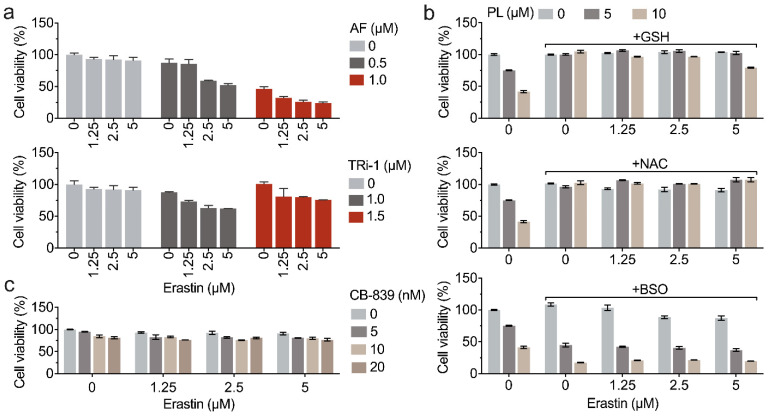
Pharmacological inhibition of TXNRD1 sensitizes cancer cells to GSH depletion. (**a**) Inhibition of TXNRD1 by auranofin (AF) or TRi-1 enhances erastin-induced cancer cells death. HCT116 cells were treated with the indicated concentrations of auranofin or TRi-1 for 5 h for inhibiting the cellular TXNRD1. After that, the medium was replaced with fresh one. The cultured cells were added with erastin at different concentrations and continually incubated for 24 h. (**b**) GSH and NAC mitigate the cytotoxicity of erastin in combination with piperlongumine and BSO enhances the lethality. HCT116 cells were co-treated with erastin at the indicated concentrations and 1 mM NAC or 1 mM GSH for 24 h. (**c**) GSL inhibitor CB-839 slightly promotes erastin-induced cell death. HCT116 cells were co-treated with the indicated concentrations of CB-839 and erastin for 24 h. The cell viabilities in panels (**a**–**c**) were determined by the CCK-8 assay.

We next sought to know whether the synergistic effects of piperlongumine and erastin were attributed to targeting TXNRD1. Using two TXNRD1 inhibitors, auranofin and TRi-1, the pharmacological inhibition of TXNRD1 was found to enhance erastin-induced cell death (Figure 6a). In cellular GSH synthesis, TXN1 and TRP14 are involved in the reduction of cystine with the help of TXNRD1 [16]. Therefore, we wanted to investigate how the cellular GSH level affects erastin-induced cell death, as the main function of erastin is to inhibit the cystine transporter SLC7A11. As shown in Figure 6b, GSH and NAC mitigated the erastin-induced cell death, as well as the combined treatment with piperlongumine, and 100 μM BSO enhance the cytotoxicity of erastin. Interestingly, the GLS inhibitor CB-839, slightly promoted the cytotoxicity of erastin. GLS catalyzes the hydrolysis of glutamine to produce glutamate, which is an important step for GSH synthesis [30,31,32]. However, pharmacological inhibition of GSL by CB-839 only slightly strengthened the cell death (Figure 6c). 

## 4. Discussion

A growing body of evidence suggests that stimulating ROS production and accumulation has the potential to be an effective chemotherapeutic strategy in treating cancers [33]. Since the predominant role of selenoprotein TXNRD1 in quenching cellular ROS, a slew of small molecule inhibitors was screened and developed to target TXNRD1, including piperlongumine [34,35,36]. Piperlongumine was first identified as selectively toxic to cancer cells rather than non-transformed cells by targeting glutathione S-transferase (GST) both in vitro and in vivo, and further demonstrated the depletion of cellular GSH [37]. Afterwards, the anti-tumor capacity of piperlongumine was tightly correlated to cellular redox homeostasis. Therefore, there is a need to disclose the interaction between piperlongumine and critical antioxidant enzyme(s) [38].

By testing the inhibitory effect of piperlongumine on TXNRD1, piperlongumine was found to irreversibly inhibit TXNRD1 activities in a dose-dependent manner. The inhibition mechanism of piperlongumine on TXNRD1 was consistent with other TXNRD1 inhibitors, such as gambogic acid, parthenolide, etc. [39,40]. The electrophilic moiety of piperlongumine modified the surface-exposed Sec residues through the Michael addition reaction. However, two potential sites of piperlongumine, the C2-C3 olefin and the C7-C8 olefin, may attack TXNRD1. Since the C2-C3 olefin is the key pharmacophore of piperlongumine and is critical for its ROS production activity [37], it was speculated that the modification of TXNRD1 by piperlongumine depends mainly on the C2-C3 olefin, but this speculation should be subjected to qualitative and structural analyses to study further.

Furthermore, we demonstrated that piperlongumine modified the Sec^498^ or Cys^498^ of TXNRD1 by using TXNRD1 mutants as shown in Figure 3. However, it cannot be ignored that the truncated (GC), GCSG, and GSCG mutants of TXNRD1 remain low turnover in reducing DTNB or other TXNRD1 substrates, which makes it restricted to confirm the certain targeted residues of piperlongumine to TXNRD1.

We also showed that piperlongumine converted TXNRD1 from an anti-oxidant to a pro-oxidant NADPH oxidase by SecTRAPs, which formed from the Sec^498^ residue of TXNRD1 targeted with electrophiles (such as cisplatin, juglone, and piperlongumine in this study). SecTRAPs may act as a critical manner of piperlongumine in causing cellular oxidative stress [41,42]. TXNRD1 is physiologically involved in diverse cell processes, such as transmitting electrons to TXN1, repairing oxidized thiol of PTP1B, opposing apoptotic by inhibiting ASK-1, and supporting DNA synthase via reducing RNR [43,44,45,46], and these cellular functions of TXNRD1 all point to cancer cells growth and proliferation.

**Figure 7:**
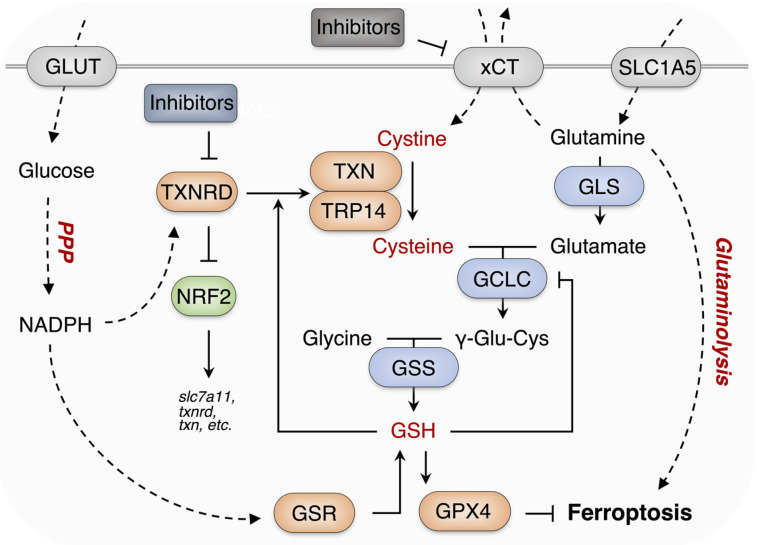
The proposed mechanism of pharmacological inhibition of TXNRD1 boosts the cytotoxicity of erastin in cancer cells. In mammals, TXN system and GSH system synergistically reduce cystine to cysteine, which is further used to form antioxidants GSH. So, pharmacological inhibition of TXNRD1 may confer cancer cells more sensitivity to GSH depletion or Cys starvation.

Pharmacological inhibition of the suppressors of ferroptosis leads to cancer cell ferroptosis [15,47]. However, some cancer cells, such as KEAP1-mutant NSCLC cells, are insensitive to ferroptosis due to the robust endogenous antioxidant activity [48,49]. A549 cells harbored abnormal activation of NRF2, due to the functional loss of KEAP1 caused by mutation and are insensitive to erastin or other ferroptosis inducers. TXNRD1 is involved in antioxidant defense and is correlated to cancer cells’ drug resistance. We, therefore, wanted to investigate whether it is possible to overcome ferroptosis resistance in cancer cells via inhibiting TXNRD1 by piperlongumine. Fortunately, piperlongumine was found to enhance erastin-induced cell death as shown in Figure 5. The synergistic effects and the underlying mechanism can easily be correlated with the cellular antioxidant TXN system, especially selenoprotein TXNRD1. SLC7A11 and TXNRD1 have been reported to cooperate to protect cells from glutathione deficiency [16]. Therefore, inhibition of SLC7A11 together with the inhibition of TXNRD1 predisposes cancer cells to “disulfide stress”, and further induces cell death [50,51]. The proposed mechanism is shown in Figure 7.

Previously, selenoprotein TXNRD1 has been found to cooperate with selenoprotein GPX4 to protect cells together from the lethal accumulation of lipid peroxides [47,52]. The TXN system was thought to be a negative regulator of iron toxicity considering its role in cells, such as cystine reduction and ROS quenching. However, genetic depletion of TXNRD1 results in increased levels of GPX4 protein, which confers protection from ferroptosis induced by small-molecule inhibition of GPX4 [52], indicating that the role of the TXN system in ferroptosis is still not clear. What is more, published studies show the survival of diverse cancer cell types that lack TXNRD1 genetically, but pharmacological inhibition of TXNRD1 induced robust cancer cell apoptosis [53,54]. Therefore, the function of TXNRD1 in programmed cell death still needs further investigation.

## 5. Conclusions

Taken together, this study demonstrated that piperlongumine inhibits TXNRD1 activity by targeting the Sec residues and converts the enzyme into pro-oxidative NADPH oxidase, resulting in rapid ROS-dependent cell death. Meanwhile, piperlongumine sensitizes cancer cells to erastin, which underscores the predominant roles of TXNRD1 in cancer cell ferroptosis.

## Data Availability

The data presented in this study are available in article.
